# The proximate composition, amino acid profile, fatty acid content, and mineral content of scale flour from three fish species as potential feeds for fish fry

**DOI:** 10.12688/f1000research.141304.2

**Published:** 2023-11-07

**Authors:** Hafrijal Syandri, Azrita Azrita, Ainul Mardiah, Netti Aryani, Andarini Diharmi

**Affiliations:** 1 Faculty of Fisheries and Marine Science, Universitas Bung Hatta, Padang, West Sumatera, 25133, Indonesia; 2 Faculty of Science and Technology, Universitas Nahdlatul Ulama Sumatera Barat, Padang, West Sumatera, 25136, Indonesia; 3 Faculty of Fisheries and Marine, Universitas Riau, Pekanbaru, Riau, 28293, Indonesia

**Keywords:** Fish scale flour, chemical composition, amino acids, mineral content, fatty acid profile

## Abstract

**Background:**

Fish scale waste is highly valued both as a functional food ingredient and a potential feed source for farmed fish. This study aimed to analyse the chemical composition, fatty acid profile, and mineral content in fish scale flour of *Osphronemus (O) goramy*, *Cyprinus (C) carpio*, and *Oreochromis (O) niloticus* as potential feed for fish fry.

**Methods:**

Fish scales were cleaned with 10% w/v NaCl solution at a ratio of 1:10 (w/w) for 24 hours at 4 °C. Agitation was used every eight hours to remove excess protein. Fish scales were evenly arranged in a cooker and cooked at 121 °C for 10 minutes with 15 *psi *pressure. After cooking, 100 grams of wet fish scales was dried at 50 °C for four hours. Dried fish scales were transformed into flour for proximate composition analysed via standard AOAC method, amino acid and fatty acid assessment employing HPLC and GC-MS, while mineral content was determined using AAS.

**Results:**

The examined fish scale flour from three species displayed significant variations in chemical components, amino acids, and minerals (p<0.01). Crude protein content spanned 49.52% to 72.94%, and fat content ranged from 0.11% to 0.23%. Magnesium levels varied between 767.82 mg/kg and 816.50 mg/kg, calcium content ranged from 3.54 mg/kg to 12.16 mg/kg, iron content was within 40.46 mg/kg to 44.10 mg/kg, and zinc content ranged from 45.80 mg/kg to 139.19 mg/kg. Predominantly, glycine emerged as the main free amino acid (FAA), varying from 13.70% to 16.08%, while histidine had the lowest content, at 0.39% to 0.71%. Conversely, fatty acid content was low in all species examined ranging from 6.73% to 9.48%.

**Conclusions:**

Flour from three farmed fish types has potential for fish fry feed due to its chemical composition, amino acid, and mineral content. Further validation is needed for amino acid comparison to fish meal.

## Introduction

According to the Food and Agriculture Organization of the United Nations (FAO), global fish production in 2018 reached approximately 179 million tonnes, with aquaculture contributing 46% of the total production. ^1^ In Indonesia, the total aquaculture production was recorded at 16,032,122 metric tonnes (mt). Of this, 3,374,924 mt (21.05%) originated freshwater aquaculture production; 9,884,670 mt (61.65%) from marine water aquaculture production, including seaweeds in general; and 2,772,568 mt (17.29%) from brackish water aquaculture production (CDSI; Central Data System Information). ^2^ Approximately 10% of global fish production is currently discarded, while byproducts from fisheries constitute 70% of the total weight of fish production. Among these byproducts, fish bones and scales constitute 14 to 20% of the total weight, and these materials are also discarded. ^3^

This significant quantity of byproducts is occasionally utilized as animal feed, fishmeal, oil, or plant fertilizer, but in most instances, it is discarded. ^4^^,^^5^ In recent years, there has been a growing recognition of environmental sustainability and a heightened emphasis on harnessing the value of resources within green and blue economies. ^6^^,^^7^ As a result, fish byproducts, including fish scale waste, ^3^^,^^8^ have gradually been applied as raw materials for human consumption. ^9^

Fish scales contain approximately 41-45% organic components, such as collagen, fat, lecithin, sclerotin, and vitamins, and 38-46% inorganic components and mineral elements, including magnesium, iron, zinc, calcium, and vitamins. ^10^ Furthermore, fish scales possess antioxidant and antihypertensive properties. ^11^ Fish scales have been used as a culinary ingredient in baked bread, ^3^ because they are a source of food that is rich in nutrients. ^6^^,^^7^ These components are also crucial for the growth and survival of fish fry. In recent years, fish fry feed has primarily been live feed, and expensive artificial feed is a bottleneck in aquaculture ^12^^,^^13^ Therefore, there is a technological gap in exploring alternative ingredients for fish fry feed, such as fish scale flour, which is a rich source of nutrients, has economic value, and provides an element of novelty in this study.

A significant quantity of fish scale waste is readily accessible in the Indonesian market, encompassing fish scale waste derived from the death of farmed fish in Lake Maninjau. ^14^ Regrettably, in the past decade, this waste has remained untapped as both a food source and an ingredient for fish fry feed. This research aims to assess the proximate, amino acid, fatty acids and mineral contents in freshwater fish scale flour, specifically flour from the scales of giant gourami ( *Osphronemus (O) goramy*), carp ( *Cyprinus (C) carpio*), and tilapia ( *Oreochromis (O) niloticus*), with the potential to be utilized as feed ingredients for fish fry.

## Methods

### Ethical considerations

The research was approved by the Research and Community Service Ethics Committee at Universitas Bung Hatta with an approval letter No.057a/LPPM/Hatta/VI-2023 dated June 23, 2023. Experiments were carried out in accordance with the guidelines outlined in the Standard Operating Procedure of Laboratory Aquaculture at Universitas Bung Hatta.

### Biometric measurement of fish sample

Ten *O. goramy*, *C. carpio*, and *O. niloticus* fish were obtained from a local fish market in Lake Maninjau, Indonesia. The fresh fish were carefully stored on ice and promptly transported to the laboratory.

Upon reaching the laboratory, the fish were individually weighed (TW) using AD-600i scales with a precision of 0.001 grams and measured to their standard length (SL) and maximum height (H), with distance measured from the mouth to the end of the upper lobe of the caudal fin and height measured vertically, excluding the fins. ^15^ Standard length and height were assessed using a meter ruler with an accuracy of 1 millimeter. The condition factor (CF) was calculated using the formula CF = (TW/SL ^3^) × 100. ^16^

### Preparation of fish scales

Upon arrival at the laboratory, the fish scales from *O. goramy*, *C. carpio*, and *O. niloticus* were collected from each fish for further processing. Fish scales were collected with a stainless steel fish scaler cleaner of 17.5 cm × 3.5 cm × 1.5 cm.

**Figure 1.**
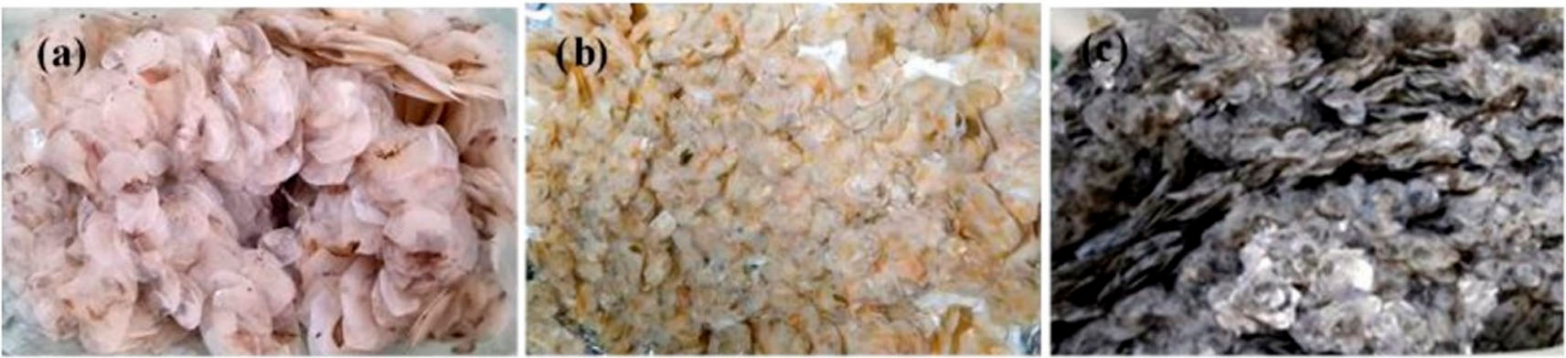
Scales of Giant gourami (a), Common carp (b), and Tilapia (c) used in this study.

Every 1,000 grams of wet scales of *O. goramy*, *C. carpio*, and *O. niloticus* were washed thoroughly to obtain 200 grams of dry scales. The fish scales were washed in a three-litres plastic jar with 10% w/v NaCl solution with a solution ratio of 1:10 (w/w). The washing procedure was conducted for a duration of 24 hours at a temperature of 4 °C. The washing process was repeated every eight hours to further improve the effectiveness of protein removal. This frequent repetition helped ensure that any remaining unnecessary proteins on the fish scales were thoroughly removed. ^3^^,^^10^ By repeating the washing process at regular intervals, the purity of the fish scales was enhanced, preparing them for further processing ( Figure 1).

Furthermore, scales of *O. goramy*, *C. carpio*, and *O. niloticus* were washed three times in low mineral-content water (with a TDS of around 100 mg/L) at room temperature for 10 minutes and then drained. Subsequently, the scales were evenly arranged in a cooker that was equipped with a pressure control button and cooking time settings (Model: Classic Pressure Cooker, with a ø 20 cm, 5.5-litre capacity, named Culinart, Made in China). The heating process was carried out using a cooker until the temperature reached 121 °C with a pressure of 15 *psi*, as indicated by the temperature and pressure panel. At this point, the timer was set for 10 minutes. The cooking time was calculated as the time between when the pressure in the cooker reached 15 *psi* and that when the heat source for cooking was turned off.

**Figure 2.**
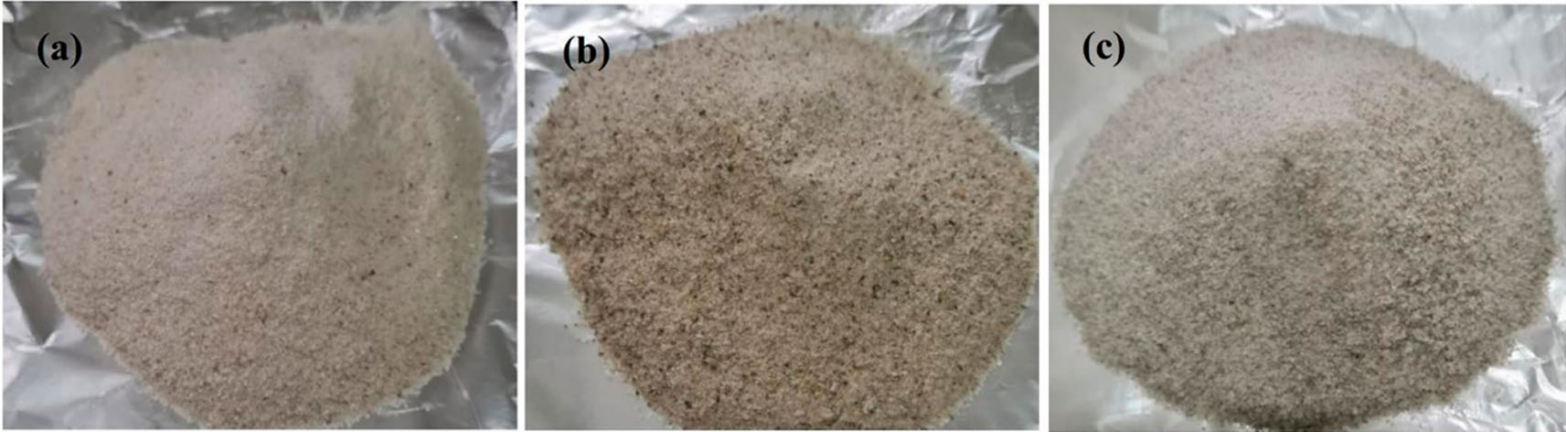
Fish scale flour from Giant gourami (a), Common carp (b), and Tilapia (c) was examined in this study.

A total of 200 grams of scales from each species ( *O. goramy*, *C. carpio*, and *O. niloticus* scales; wet weight) was dried using a 28 L stainless steel black digimatic oven tester at 50 °C for four hours until the moisture content reached 10%. The dried fish scales were then processed into flour using a Miller Powder Grinder with a 100-gram capacity. The resulting flour was then sieved using a mesh size of 60 μm to analyse the proximate composition and amino acid, fatty acid, and mineral contents ( Figure 2).

### Proximate and amino acid composition

The proximate composition of the fish scale samples was analysed using standard AOAC methods. ^17^ The samples were dried at 105 °C until a constant weight was achieved. The crude protein content was analysed using the standard Kjeldahl method, calculated as N × 6.25. Crude lipids were analysed using the Soxhlet method with ether extraction. The ash content was determined by incinerating the samples at 550 °C for 16 hours. Gross energy was measured using a bomb calorimeter.

Total carbohydrates were determined by subtracting the sum of % crude protein (CP), % fat (F) and % ash contents (A) from 100 ^18^ by using the following equation: % Total carbohydrates = 100 – (CP + F + A). The gross energy value of each sample was determined by augmenting the percentage of crude protein (CP), fat (F), and total carbohydrate (C) contents with their respective energy values of 4, 9, and 4 kcal per 100 g of scale flour, respectively, to obtain the caloric values of the samples by using the following equation= (4CP + 9F + 4C)kcal/100 g weight.

The methods described by Ref. 19 were employed for amino acid analysis. The amino acid composition was determined using a high-performance liquid chromatography (HPLC) system, which consisted of a Waters 1525 binary HPLC pump, 717 autosamplers (Waters ^®^), and Waters 2475 multi λ fluorescence detector optics (with wavelengths set at 250 nm for excitation and 395 nm for emission). The samples were hydrolysed in triplicate using 6 N hydrochloric acid for 24 hours at 11 °C.

### Mineral content analysis

For the analysis of mineral content (Na, Mg, Ca, K, P, Fe, and Zn), the ashed GCTS sample was dissolved in 1 ml of hydrochloric acid (35% v/v Suprapur ^®^ Merck). Subsequently, the sample was filtered using cellulose filter paper (Watchman No 1, International Ltd; Maidstone, UK) and appropriately diluted for each elemental mineral and finally analyse with a Perkin-Elmer AA mod 3110 spectrophotometer (Norwalk, CT, USA). ^20^ Phosphorus (P) levels were analysed using a Perkin-Elmer AA spectrophotometer mod 3110 (Norwalk, CT, USA). ^20^

### Fatty acid analysis

The fatty acid composition of fish scale flours was examined through gas chromatography-mass spectrometry (GC-MS) analysis. The total lipid extraction followed a modification of the method by Folch *et al*. (1957), as detailed by Rajion, ^21^ employing a chloroform: methanol (2.1, v/v) solvent system. Transmethylation was carried out with 14% methanolic boron trifluoride.

### Data analysis

Data analysis was conducted using Statistical Package for the Social Sciences ( SPSS) 16.0 software (SPSS; Chicago, IL). The homogeneity of the data was assessed using the Levine test. One-way ANOVA was performed to determine the proximate and amino acid composition parameters and the mineral content for each fish scale flour of the three species. *Post hoc* analysis was carried out using Duncan’s multiple-range test. ^22^ The results are reported as the mean values ± standard errors for each parameter.

## Results and discussion

### Biometric measurement

Table 1 displays the average standard length, wet weight, height, condition factor, and chemical composition of three fish species found in Lake Maninjau. Statistically significant variations were noted in the standard length, wet weight, height, and condition factor of the three examined fish species (p < 0.05; Table 1). ^52^

**Table 1.** Biometric, proximate composition and mineral content of scale flour of three fish species.

	Species			
	*O.goramy*	*C. carpio*	*O. niloticus*	*α*
*Biometric measurements*				
Wet weight (g)	595.40 ± 32.31 ^a^	633.30 ± 87 ^b^	210.5 ± 12.12 ^c^	***
Standard length (cm)	23.32 ± 0.62 ^a^	26.01 ± 2.03 ^b^	18.0 ± 0.53 ^c^	***
Height (cm)	12.60 ± 0.36 ^a^	9.90 ± 0.44 ^b^	7.15 ± 0.21 ^c^	***
Condition factor	3.91 ± 0.22 ^a^	3.99 ± 0.65 ^b^	3.61 ± 0.21 ^c^	***
*Proximate composition*				
Moisture % of dry weight	6.74 ± 0.01 ^a^	7.41 ± 0.03 ^b^	5.74 ± 0.02 ^c^	***
Crude protein (%)	56.44 ± 0.02 ^a^	72.94 ± 0.10 ^b^	49.52 ± 0.01 ^c^	***
Fat (%)	0.13 ± 0.00 ^a^	0.23 ± 0.02 ^b^	0.11 ± 0.00 ^c^	***
Ash (%)	32.71 ± 0.58 ^a^	15.45 ± 0.15 ^b^	40.31 ± 0.03 ^c^	***
Fibre (%)	1.22 ± 0.03 ^a^	1.38 ± 0.01 ^b^	1.32 ± 0.02 ^c^	***
Total carbohydrates (%)	11.01 ± 0.55	11.56 ± 0.18	10.90 ± 0.06	ns
Energy value (kkal/100 g DM)	271.05 ± 2.36 ^a^	339.35 ± 0.50 ^b^	239.13 ± 0.16 ^c^	***
*Mineral composition*				
Macro mineral (mg/kg)				
Sodium (Na)	2,828.13 ± 0.87 ^a^	6,196.65 ± 0.30 ^b^	10,748.66 ± 0.13 ^c^	***
Magnesium (Mg)	816.50 ± 0.21 ^a^	767.82 ± 0.10 ^b^	794.79 ± 0.20 ^c^	***
Calcium (Ca)	10.81 ± 0.03 ^a^	3.54 ± 0.06 ^b^	12.16 ± 0.03 ^c^	***
Potassium (K)	2,111.29 ± 0.11 ^a^	133.88 ± 0.10 ^b^	252.84 ± 0.10 ^c^	***
Phosphorous (P)	6.15 ± 0.02 ^a^	2.74 ± 0.02 ^b^	7.33 ± 0.05 ^c^	***
Microminerals (mg/kg)				***
Iron (Fe)	44.10 ± 0.05 ^a^	40.46 ± 0.09 ^b^	41.52 ± 0.15 ^c^	***
Zinc (Zn)	45.80 ± 0.21 ^a^	139.19 ± 0.05 ^b^	55.43 ± 0.04 ^c^	***

Mean ± SD (n = 3) with different letters in the same row are significantly different.

Level of significance (α):

*** p < 0.001; ns: non-significant.

### Proximate composition and mineral content

In general, there was a significant difference (p < 0.05; Table 1) in the proximate content of fish scale flour between the three farmed fish species in Lake Maninjau. The water content, crude protein content, fat content, and energy values were higher in *C. carpio* scale flour than in *O. goramy* and *O. niloticus* scale flours ( Table 1).

The highest protein content was relatively recorded in the fish scale flour of *C. carpio*, and the content did not differ by more than 23% between the three fish groups. Huang *et al*. ^23^ reported that tilapia fish scale flour contained 49.42% protein, 0.02% lipid, 45.18% ash, and 5.38% carbohydrates on a dry weight basis. Similarly, the protein content of spotted golden goatfish ( *Parupeneus heptacanthus*) was 45.2%. ^24^ On the other hand, protein valuation from demineralized fish scale gelatine and nondemineralized gelatine displayed protein purities of 57.19 g/100 g and 43.37 g/100 g, respectively. ^3^

Higher fat levels of *C. carpio* scale flour (0.23%) than in *O. goramy* (0.13%) and *O. niloticus* (0.11%) scale flour have also been observed for *Labeo rohita* ^25^ and other species. ^26^^,^^27^ This result could be due to various factors, including availability and dietary protein intake, fish size and age, and fish scale type. ^23^^,^^25^

Furthermore, there were significant differences in the mineral content of fish scale flour between species (p <0.05; Table 1). The magnesium, potassium, and iron levels were higher in the *O. goramy* fish scale flour than in the other scale flour samples. At the same time, the sodium, calcium, and phosphorous contents were higher in the *O. niloticus* fish scale flour. Moreover, zinc levels were higher in *C. carpio* fish scale flour ( Table 1) than in the other scale flour samples. In general, the levels of the minerals analysed in this study are in alignment with the results of previous studies. ^28^^,^^29^

The flour derived from fish scales of three farmed fish species exhibited a high mineral content, making it a potentially suitable choice for utilization as feed for fish fry. The inclusion of these minerals in fish feed is crucial because they serve as essential nutrients for the nourishment of fingerlings. As stated by Nagappan *et al*., ^30^ fibre, minerals, and vitamins are essential, albeit minimal, requirements for optimal fish growth performance. In the context of fish scale flour, it was observed that all three species contained elevated mineral levels, implying that the scale flour offers enhanced support for the growth of fish fry. Nevertheless, it is important to highlight that despite the relatively high mineral content in the feed, there was no significant impact observed on the development of experimental animals, as noted by Dominquez *et al*. ^31^ and Wang *et al*. ^32^

### Free amino acids (FAAs)

The FAA profiles for the scale flour of the three fish species are presented in Table 2. Statistically significant differences were recorded in the FAA for the scales of the three fish species studied (p < 0.05; Table 2). The three species of farmed fish showed higher levels of aspartic acid, glycine, and alanine and lower levels of serine, histidine, methionine, and isoleucine ( Table 2). *C. carpio* scale flour of showed the highest total FAA content (62.74%) compared to that of farmed fish ( *O. goramy*; 48.31% and *O. niloticus*; 41.58%). The differences in the FAA profile could be related to different aspects, such as fish species, wild or farmed fish origin, diet composition, feeding habits, animal size, and age. ^33^^–^^36^

**Table 2.** Profile of free amino acids in scale flours from three fish species (% ww/ww).

	Scales flour			
	*O. goramy*	*C. carpio*	*O. niloticus*	α
Aspartic acid	2.93 ± 0.04 ^a^	4.21 ± 0.03 ^b^	2.59 ± 0.01 ^c^	***
Glutamic acid	6.19 ± 0.01 ^a^	8.03 ± 0.01 ^b^	5.28 ± 0.01 ^c^	***
Serine	1.91 ± 0.01 ^a^	2.85 ± 0.01 ^a^	1.76 ± 0.01	***
Glycine	16.08 ± 0.01 ^a^	19.08 ± 0.01 ^b^	13.70 ± 0.01 ^c^	***
Histidine	0.39 ± 0.01 ^a^	0.71 ± 0.01 ^b^	0.42 ± 0.01 ^c^	***
Arginine	3.67 ± 0.01 ^a^	5.15 ± 0.01 ^b^	3.19 ± 0.01 ^c^	***
Threonine	1.50 ± 0.03 ^a^	2.04 ± 0.01 ^b^	1.34 ± 0.01 ^c^	***
Alanine	6.06 ± 0.01 ^a^	6.97 ± 0.00 ^b^	5.01 ± 0.01 ^c^	***
Tyrosine	1.79 ± 0.01 ^a^	2.29 ± 0.01 ^b^	1.51 ± 0.01 ^c^	***
Valine	1.53 ± 0.00 ^a^	1.89 ± 0.01 ^b^	1.24 ± 0.01 ^c^	***
Methionine	0.37 ± 0.01 ^a^	1.45 ± 0.01 ^b^	0.50 ± 0.02 ^c^	***
Isoleucine	0.91 ± 0.01 ^a^	1.46 ± 0.00 ^b^	0.76 ± 0.01 ^c^	***
Leucine	0.90 ± 0.01 ^a^	2.34 ± 0.01 ^b^	1.46 ± 0.01 ^c^	***
Phenylalanine	1.34 ± 0.01 ^a^	1.99 ± 0.01 ^b^	1.23 ± 0.01 ^c^	***
Lysine	2.03 ± 0.01 ^a^	2.30 ± 0.01 ^b^	1.57 ± 0.01 ^c^	***
Total	48.31	62.74	41.58	

Mean ± SD (n = 3) with different letters in the same row are significantly different.

Level of significance (α):

*** p < 0.001.

In all samples, glycine was the most abundant FAA, which is in accordance with other studies on fish scale collagen in tilapia, *Oreochromis* sp., ^23^ and the whole-body carcass of *Hemibagrus nemurus* ^37^ and *O. goramy.* ^38^ Glycine has been reported to be one of the essential components in the collagen molecule, helping maintain tissue strength and elasticity. ^39^^,^^40^

Following glycine, the most abundant FAAs were glutamic acid, alanine, and arginine. Furthermore, histidine, methionine, and isoleucine were present in all samples but in smaller quantities; these particular FAA are commonly found in greater proportions within aquatic organisms. ^41^^,^^42^

### Fatty acid profile

The fatty acid composition in the fish scale flour of the three cultivated fish species is displayed in Table 3. This discovery is in line with data previously reported for Atlantic salmon ( *salmo salar*) and Catla catla ( *Labeo catla*), ^43^^,^^44^ where the fatty acid content of both species is relatively low. Variations in the fatty acid composition were evident among the three cultured fish species, with statistically significant differences (p < 0.05) observed among the species. Nevertheless, only two out of the fourteen saturated fatty acids (SAFAs) were identified. In monounsaturated fatty acids (MUFAs), three out of eight were observed, whereas three of the ten polyunsaturated fatty acids (PUFAs) were found. This result is in contrast to the type of fatty acids detected in the carcasses of several species of fish. ^45^^–^^48^

**Table 3.** Fatty acids concentrations of scale flours from three fish species (%w/w).

	Fish scales flour			
	*O. goramy*	*C. carpio*	*O. niloticus*	α
*Saturated fatty acids (SAFAs)*				
Butyric Acid, C4:0	0.00	0.00	0.00	
Caproic acid, C6:0	0.00	0.00	0.00	
Caprilic acid, C8:0	0.00	0.00	0.00	
Capric acid, C10:0	0.00	0.00	0.00	
Undecanoic acid, C11:0	0.85 ± 0.01 ^a^	0.58 ± 0.01 ^b^	0.72 ± 0.01 ^c^	***
Lauric Acid, C12:0	0.00	0.00	0.00	
Tridecanoic Acid, C13:0	0.40 ± 0.01 ^a^	0.36 ± 0.01 ^b^	0.17 ± 0.01 ^c^	***
Myristic Acid, C14:0	0.00	0.00	0.00	
Pentadecanoic Acid, C15:0	0.00	0.00	0.00	
Palmitic Acid, C16:0	0.00	0.00	0.00	
Heptadecanoic Acid, C17:0	0.00	0.00	0.00	
Stearic Acid, C18:0	0.00	0.00	0.00	
Arachidic Acid, C20:0	0.00	0.00	0.00	
Behenic Acid, C22:0	0.00	0.00	0.00	
Total SAFAs	1.25	0.94	0.89	
*Monounsaturated fatty acids (MUFAs)*				
Myristoleic Acid, C14:1	0.00	0.00	0.00	
Cis-10-Pentadecanoic Acid, C15:1	2.02 ± 0.01 ^a^	2.01 ± 0.01 ^b^	2.26 + 0.01 ^c^	***
Palmitoleic Acid, C16:1	0.00	0.00	0.00	
Cis-10-Heptadecanoic Acid, C17:1	0.73 ± 0.01 ^a^	1.21 ± 0.01 ^b^	1.19 ± 0.01 ^c^	***
Elaidic Acid, C18:1n9t	2.09 ± 0.01 ^a^	2.28 ± 0.01 ^b^	3.71 ± 0.02 ^c^	***
Oleic Acid, C18:1n9c	0.00	0.00	0.00	
Erucic Acid Methyl Ester, C22:1n9	0.00	0.00	0.00	
Nervonic Acid, C24:1	0.00	0.00	0.00	
Total MUFAs	4.84	5.50	7.16	
*Polyunsaturated fatty acids (PUFAs)*				
Linolelaidic Acid, C18:2n9	0.00	0.00	0.00	
Linoleic Acid, C18:2n6c	0.15 ± 0.01 ^a^	0.12 ± 0.01 ^b^	0.17 ± 0.01 ^c^	***
v-Linolenic Acid, C18:3n6	0.22 ± 0.00 ^a^	0.28 ± 0.01 ^b^	0.40 ± 0.01 ^c^	***
Linolenic Acid, C18:3n3	0.00	0.00	0.00	
Cis-8,11,14-Eicosetrienoic Acid, C20:3n6	0.00	0.00	0.00	
cis-11, 14, 17-Eicosatrienoic Acid Methyl Ester, (C20:3n3)	0.00	0.00	0.00	
Arachidonic Acid, C20:4n6	0.00	0.00	0.00	
Cis-13,16-Docosadienoic Acid, C22:2	0.00	0.00	0.00	
Cis-5,8,11,14,17-Eicosapentaenoic Acid, C20:5n3	0.27 ± 0.01 ^a^	0.48 ± 0.01 ^b^	0.86 ± 0.01 ^c^	***
Cis-4,7,10,13,16,19-Docosahexaenoic Acid, C22:6n3	0.00	0.00	0.00	
Total PUFAs	0.64	0.88	1.43	
∑ Fatty acid	6.73	7.32	9.48	

Mean ± SD (n = 3) with different letters in the same row are significantly different.

Level of significance (α):

*** p < 0.001.

Fish scale flour from *O. niloticus* exhibited the highest cumulative fatty acid content (9.48%) in comparison to farmed fish scale flour ( *C. carpio*; 7.32% and *O. goramy*; 6.73%). These findings are very similar in structure to the results obtained from analysis of the composition of saturated fatty acids (SFAs), monounsaturated fatty acids (MUFAs), and polyunsaturated fatty acids (PUFAs) in the whole-body carcass of giant gourami and other fish species. ^33^^,^^35^^,^^46^

Based on the analysis of the composition of the fatty acids contained in the scale flour of three species of farmed fish, if it is intended as feed for fish fry, it is necessary to enrich this fish scale flour with compounds containing fatty acids. Some enrichment options to consider are fish oil, chia seed oil, flaxseed oil, and walnut oil. These four sources are rich in omega-3 fatty acids, especially eicosapentaenoic acid (EPA) and docosahexaenoic acid (DHA), as well as alpha-linolenic acid (ALA). Fish scale flour from three types of farmed fish can be validated for amino acids, fatty acids, and minerals for fish fry feed or as a substitute for fish meal and soybean meal. Rodrigues et al ^49^ showed that amino acids such as lysine increased the growth of juvenile pacu, *Piaractus mesopotamicus*. In contrast, fatty acids, such as linolenic acid, EPA, and DHA, are required in commercial feeds to meet the physiological, growth, and health needs of largemouth bass, *Micropterus salmoides*. ^50^ In addition, minerals such as Na, Mg, K, Cu, Fe, and Zn are very important for optimizing the growth of Largemouth bass, *Micropterus salmoides*. ^51^ These examples show the influence of various amino acids, fatty acids and minerals in feed when cultivating various fish species. They also highlighted the need for unique feed formulations to improve fish survival and growth performance.

## Conclusion

Fish scale flour derived from *O. goramy*, *C. carpio*, and *O. niloticus* in the study region was identified as a valuable protein, amino acid, and mineral source. All fish scale flour samples across the three species contained amino acids and minerals but low fatty acids. Consequently, this needs to be validated in the context of amino acid requirements with fish meal or other ideal protein sources that meet the requirements for the cultured species. There is a lack of reliable data regarding the chemical composition, mineral, and fatty acid profiles of fish scale flours from the three local fish species in the study area. Therefore, the chemical, mineral, and fatty acid composition data presented in this study will be the groundwork for future research in fish scale flour chemistry, contributing to fish fry nutrition optimization.

## Data Availability

Figshare: The Proximate Composition, Amino Acid Profile, Fatty Acid Content, and Mineral Content of Scale Flour from Three Fish Species as Potential Feeds for Fish Fry, https://doi.org/10.6084/m9.figshare.23954799. ^52^ This project contains the following underlying data:
-
Table 1. Raw biometric data of three species of farmed fish samples. -
Table 2. Raw proximate composition data of fish scale flour from three Species (%W/W). -
Table 3. Raw mineral content data of fish scale flour from three Species (mg/kg). -
Table 4. Raw Amino Acid Composition Data of Fish Scale Flour from Three Species (%W/W). -
Table 5. Raw fatty Acid Composition Data of Fish Scale Flour from Three Species (%W/W). Data are available under the terms of the Creative Commons Attribution 4.0 International license (CC-BY 4.0).
