# Supplementary material for: The proximate composition, amino acid profile, fatty acid content, and mineral content of scale flour from three fish species as potential feeds for fish fry
Source: F1000Res. 2023 Nov 7;12:1144. Originally published 2023 Sep 13. [Version 2] doi: 10.12688/f1000research.141304.2 (PMC10733663; doi:10.12688/f1000research.141304.2)
Supplement: Peer review history

# Reviewer report: Reviewer response for version 2

**DOI:** 10.5256/f1000research.158365.r221453

**Published:** 2023-12-20

**Authors:** Adhita Sri Prabakusuma

**Affiliations:** 1 Vocational School of Foodservice Industry, Food Biotechnology Research Group, Universitas Ahmad Dahlan, Yogyakarta, Indonesia

I apologize for the delay in the review process, as I completed our year-end research projects and lecture commitments. After careful review and thorough verification, I have checked several sections of this revised version. I noticed significant improvements made by the authors. For instance, the authors have included in the introduction the crucial function of fish scales as a nutrient-rich food component and have thoroughly discussed the technological gaps in using fish scale flour as feed for fish fry. Additionally, the authors have appropriately updated the method section with the necessary references to assist in the biometric aspects and mineral content analyses. Furthermore, the authors have also thoroughly discussed the comparative protein levels and the importance of amino acids, fatty acids, and minerals in fish feed for enhancing the survival and growth performance of fish.

Is the work clearly and accurately presented and does it cite the current literature?

Yes

If applicable, is the statistical analysis and its interpretation appropriate?

Yes

Are all the source data underlying the results available to ensure full reproducibility?

Yes

Is the study design appropriate and is the work technically sound?

Yes

Are the conclusions drawn adequately supported by the results?

Yes

Are sufficient details of methods and analysis provided to allow replication by others?

Yes

Reviewer Expertise:

Food safety and quality, food science and technology, food waste management, and agri-food system

I confirm that I have read this submission and believe that I have an appropriate level of expertise to confirm that it is of an acceptable scientific standard.

# Reviewer report: Reviewer response for version 2

**DOI:** 10.5256/f1000research.158365.r221454

**Published:** 2023-11-27

**Authors:** Peter Vilhelm Skov

**Affiliations:** 1 DTU Aqua, Section for Aquaculture, The North Sea Research Centre, Technical University of Denmark, Hirtshals, Denmark

I have no further comments

Is the work clearly and accurately presented and does it cite the current literature?

Yes

If applicable, is the statistical analysis and its interpretation appropriate?

Partly

Are all the source data underlying the results available to ensure full reproducibility?

Yes

Is the study design appropriate and is the work technically sound?

Yes

Are the conclusions drawn adequately supported by the results?

Yes

Are sufficient details of methods and analysis provided to allow replication by others?

Partly

Reviewer Expertise:

Fish physiology, energetics and nutrition

I confirm that I have read this submission and believe that I have an appropriate level of expertise to confirm that it is of an acceptable scientific standard.

# Reviewer report: Reviewer response for version 1

**DOI:** 10.5256/f1000research.154735.r212279

**Published:** 2023-10-30

**Authors:** Peter Vilhelm Skov

**Affiliations:** 1 DTU Aqua, Section for Aquaculture, The North Sea Research Centre, Technical University of Denmark, Hirtshals, Denmark

Abstract:

 "Conversely, fatty acid content was lowest among the species, ranging from 6.73% to 9.48%." should be rephrased to "Fatt acid content was low in all species examined, ranging from 6.73% to 9.48%."

 " **Conclusions: **Scale flour from three farmed fish types showed potential for fish fry feed due to its chemical composition and amino acid and mineral contents. To enhance the essential fatty acid content, enriching the flour with oils containing eicosapentaenoic acid (EPA), docosahexaenoic acid (DHA), and α-linolenic acid (ALA) is essential"

 I don't agree with this statement. An ideal ingredient for feed formulation need not contain FA (e.g. fishmeal is also low in FA. The conclusion must be that it is a suitable protein source fir fry feed. This should however be validated in a context of AA requirement. Is the AA content comparable to fish meal or another ideal protein source, and would it satisfy the AA requirements of farmed species.

 Introduction:

 Do you means seagrass specifically, or seaweeds in general?

 Methods:

 Can you be more specific concerning "low mineral content water?"

 "Mineral content analysis

 For the analysis of mineral content (Na, Mg, Ca, K, P, Fe, and Zn), the ashed GCTS sample was dissolved in 1 ml of hydrochloric acid (35% v/v Suprapur ^®^ Merck). Subsequently, the sample was filtered using cellulose filter paper (Watchman No 1, International Ltd; Maidstone, UK) and appropriately diluted for each elemental mineral. Phosphorus (P) levels were analysed using a Perkin-Elmer AA spectrophotometer mod 3110 (Norwalk, CT, USA)."

 Phosphorous was analysed using the spectrophotometer, is there a reference for the method? How were the other minerals analysed following acid treatment? This information is missing.

 Proximate composition and mineral content:

 "The highest protein content was recorded in the fish scale flour of *C. carpio*, and the content did not differ by more than 23%" - specify whether it is 23% in relative or absolute terms - insert "absolute" before content.

 Conclusion:

 As hinted at in the abstract, I would appreciate that as part of the assessment of the value of the protein, that you consider the AA composition relative to other protein sources used for fish feeds (e.g. fish meal, soy) or against the nutritional requirements of selected farmed species (e.g. tilapia, carp)

Is the work clearly and accurately presented and does it cite the current literature?

Yes

If applicable, is the statistical analysis and its interpretation appropriate?

Partly

Are all the source data underlying the results available to ensure full reproducibility?

Yes

Is the study design appropriate and is the work technically sound?

Yes

Are the conclusions drawn adequately supported by the results?

Yes

Are sufficient details of methods and analysis provided to allow replication by others?

Partly

Reviewer Expertise:

Fish physiology, energetics and nutrition

I confirm that I have read this submission and believe that I have an appropriate level of expertise to confirm that it is of an acceptable scientific standard, however I have significant reservations, as outlined above.

# Response

**Published:** 2023-10-31

**Authors:** Hafrijal Syandri

**Affiliations:** Bung Hatta University, Indonesia

**Abstract:**

1. The authors agreed to change the sentence: "Conversely, fatty acid content was lowest among the species, ranging from 6.73% to 9.48%, to Fatty acid content was low in all species examined, ranging from 6.73% to 9.48%.
2. The author will add in the new version that scale flour from three types of cultivated fish has potential as fish seed feed because of its close composition, amino acid, and mineral content. However, this needs to be validated in the context of amino acid requirements. Is it comparable to fishmeal or other ideal protein sources that meet the amino acid requirements for cultured species.

**Introduction:**

1. Do you means seagrass specifically, or seaweeds in general? : The author will change seagrass to seaweed in general in new versions.

**Methods: **

1. Can you be more specific concerning "low mineral content water?". The author will added "low mineral-content water (with a TDS of around 100 mg/L)" in new versions.
2. Phosphorous was analysed using the spectrophotometer, is there a reference for the method?: Phosphorus (P) levels were analysed using a Perkin-Elmer AA spectrophotometer mod 3110 (Norwalk, CT, USA) (Perkin-Elmer (1982)
3. How were the other minerals analysed following acid treatment? This information is missing: The author will add new versions after the sentence dilute appropriately for each mineral element and finally analyze with a Perkin–Elmer AA mod 3110 spectrophotometer (Norwalk, CT, USA).
4. Specify whether it is 23% in relative or absolute terms - insert "absolute" before content: The author state that the highest protein content was relatively recorded in *C. carpio* fish scale flour, and the content did not differ by more than 23%.

**Conclusion:**

 In new revisions,  in the conclusion part, the author will change the sentence "Consequently, enriching fish scale flour with animal and plant oils rich in omega-3 fatty acids is vital" to "However, this needs to be validated in the context of amino acid requirements with fish meal or other ideal protein sources that meet the requirements for the cultured species."

# Reviewer report: Reviewer response for version 1

**DOI:** 10.5256/f1000research.154735.r208002

**Published:** 2023-10-16

**Authors:** Adhita Sri Prabakusuma

**Affiliations:** 1 Vocational School of Foodservice Industry, Food Biotechnology Research Group, Universitas Ahmad Dahlan, Yogyakarta, Indonesia

The manuscript, entitled “The proximate composition, amino acid profile, fatty acid content, and mineral content of scale flour from three fish species as potential feeds for fish fry,” focuses on analyzing the chemical composition, fatty acid profile, and mineral content in fish scale flour of *Osphronemus* (O) *goramy*, *Cyprinus* (C) *carpio*, and *Oreochromis* (O) *niloticus* as potential feed for fish fry, provides insights into chemical properties influencing nutritional aspects in feed formulation for fish fry, and describes the enrichment of the fish scale flour with oils containing essential fatty acids. After careful review of the manuscript, I feel that it is an interesting study supported by a significant amount of data and contains an important topic within the scope of the Agriculture, Food, and Nutrition Gateway of F1000Research. However, there are some critical concerns that require attention to improve the quality of the manuscript before it is accepted for publication. The specific comments are as follows:

- Please include a statement in the background section of the abstract about the potential of fish scale flour by-products as a potential source of farmed fish feed, not only as a food additive and a functional food ingredient, aligning it with the title and overall context of the paper.
- Please include the analysis approaches for measuring tested parameters mentioned in the methods section of the abstract, as the authors outlined: proximate was analyzed using standard AOAC methods; amino acid composition was determined using high-performance liquid chromatography (HPLC); and fatty acid composition was examined through gas chromatography-mass spectrometry (GC-MS).
- Please include the comparative study results of tested parameters (the proximate composition, amino acid profile, fatty acid content, and mineral content) of three fish species in the results section of the abstract.
- In the introduction section, it is better to elaborate on the recent research on utilizing fish scales both for human consumption and fish feed formulation and to make a gap analysis by evaluating the effectiveness or performance of previous methods or results.
- In the materials and methods section, put the relevant references to support the procedures for measuring the biometric properties of fish samples and preparing fish scales.
- Support this statement “This frequent repetition helped ensure that any remaining unnecessary proteins on the fish scales were thoroughly removed,” with the relevant references.
- Why did the authors select a temperature of 121 °C with a pressure of 15 *psi* to perform the heating process? Why not choose a higher temperature and more pressure for less than or within ten minutes?
- In the results and discussion section, it is also better to elaborate on the recent research studies and compare those with the findings of this current study.
- In all sub-sections, mention the function of chemical nutrition, amino acids, and fatty acids to support the growth of farmed fish, their effect on increasing the quality of fish carcasses, and their role in maintaining fish health.
- Please double-check the use of English grammar in the manuscript.

Is the work clearly and accurately presented and does it cite the current literature?

Yes

If applicable, is the statistical analysis and its interpretation appropriate?

Yes

Are all the source data underlying the results available to ensure full reproducibility?

Yes

Is the study design appropriate and is the work technically sound?

Yes

Are the conclusions drawn adequately supported by the results?

Yes

Are sufficient details of methods and analysis provided to allow replication by others?

Yes

Reviewer Expertise:

Food safety and quality, food science and technology, food waste management, and agri-food system

I confirm that I have read this submission and believe that I have an appropriate level of expertise to confirm that it is of an acceptable scientific standard, however I have significant reservations, as outlined above.

# Response

**Published:** 2023-10-25

**Authors:** Hafrijal Syandri

**Affiliations:** Bung Hatta University, Indonesia

**Response to comments from Adhita Sri Prabakusuma**

**Abstract:** The author could only revise the introduction and method sections in the abstract due to the maximum abstract length of 300 words. We will change in the revised version.

**Background:**  Fish scale waste is highly valued as a functional food ingredient and a potential feed source for farmed fish. This study aimed to analyse the chemical composition, amino acids, fatty acids, and mineral content in fish scale flour of Osphronemus (O) goramy, Cyprinus (C) carpio, and Oreochromis (O) niloticus as potential feed for a fish fry.

**Methods:** Fish scales were cleaned with 10% w/v NaCl solution at a ratio of 1:10 (w/w) for 24 hours at 4 °C. Agitation was used every eight hours to remove excess protein. Fish scales were evenly arranged in a cooker and cooked at 121 °C for 10 minutes with 15 psi pressure. After cooking, 100 grams of wet fish scales was dried at 50 °C for four hours. Dried fish scales were transformed into flour for proximate composition analysis via standard AOAC methods, amino acid and fatty acid assessment employing HPLC and GC-MS, while mineral content was determined using AAS.

**Results:** Examined fish scale flour from three species showed significant variations in chemical components, amino acids, and minerals (p<0.01). Crude protein content ranged from 49.52% to 72.94%, while fat content ranged from 0.11% to 0.23%. Magnesium levels varied from 767.82 to 816.50 mg/kg, calcium content from 3.54 to 12.16 mg/kg, iron from 40.46 to 44.10 mg/kg, and zinc from 45.80 to 139.19 mg/kg. Predominantly, glycine was the main free amino acid (FAA), varying from 13.70% to 16.08%, while histidine had the lowest content, 0.39% to 0.71%. Conversely, fatty acid content was lowest among the species, ranging from 6.73% to 9.48%.

**Conclusion:** Scale flour from three farmed fish types exhibits potential as fish fry feed, considering its chemical composition, amino acid, and mineral content. Enriching the flour with EPA, DHA, and ALA-containing oils is crucial to boost essential fatty acids.

**In the introductory part**

 The author will add to the new revised version a sentence highlighted in yellow in paragraph 3 of the "introduction" section to explain recent research and gaps in evaluating the effectiveness or performance of previous methods or results.

 Fish scales contain approximately 41-45% organic components, such as collagen, fat, lecithin, sclerotin, and vitamins, and 38-46% inorganic components and mineral elements, including magnesium, iron, zinc, calcium, and vitamins ^10^. Furthermore, fish scales possess antioxidant and antihypertensive properties ^11^. Fish scales have been used as a culinary ingredient in baked bread ^3^, because they are a source of food that is rich in nutrients ^5,7^.These components are also crucial for the growth and survival of fish fry. In recent years, fish fry feed has primarily been live feed, and expensive artificial feed is a bottleneck in aquaculture ^12,13^. Therefore, there is a technological gap in exploring alternative ingredients for fish fry feed, such as fish scale flour, which is a rich source of nutrients, has economic value, and provides an element of novelty in this study.

**Material and Methods Part**

 In the revised version, in the materials and methods section, the author will include relevant references to support procedures for measuring biometry properties of fish samples and preparing fish scales.

 Upon reaching the laboratory, the fish were individually weighed (TW) using AD-600i scales with a precision of 0.001 grams and measured to their standard length (SL) and maximum height (H), with distance measured from the mouth to the end of the upper lobe of the caudal fin and width measured vertically, excluding the fins (Famoofo and Abdul, 2020). Standard length and width were assessed using a meter ruler with an accuracy of 1 millimeter. The condition factor (CF) was calculated using the formula CF = (TW/SL ^3^) x 100 (Froese, 2006)

 This frequent repetition helped ensure that any remaining unnecessary proteins on the fish scales were thoroughly removed (Lou et al., 2020; Boronat et al., 2023).

 Why did the authors select a temperature of 121 °C with a pressure of 15 psi to perform the heating process? Why not choose a higher temperature and more pressure for less than or within ten minutes?

 The author ensures that at a temperature of 121°C with a pressure of 15 psi for 10 minutes, it is hoped that no damage will occur to the chemical composition of the fish scale flour to be analyzed.

**Discussion part**

 In the new revised version, we agreed to add comparisons with current research findings in the discussion section.

 The author will add to the newly revised version information regarding the function of amino acids, fatty acids, and minerals in the context of increasing fish seed growth.

**English grammar in the manuscript**

 Please double-check the use of English grammar in the manuscript: The language contained in this manuscript has been checked by the American Journal Experts (AJE) with the English version (United Kingdom English) and is accompanied by a certificate.
